# Development and validation of a nonverbal consensus-based semantic memory paradigm in patients with epilepsy

**DOI:** 10.1017/S1355617724000158

**Published:** 2024-04-15

**Authors:** Edwina B. Tran, Jet M.J. Vonk, Kaitlin Casaletto, Da Zhang, Raphael Christin, Siddharth Marathe, Maria Luisa Gorno-Tempini, Edward F. Chang, Jonathan K. Kleen

**Affiliations:** 1 Department of Neurology, University of California, San Francisco, CA, USA,; 2 Weill Institute for Neurosciences, University of California, San Francisco, CA, USA,; 3 Memory and Aging Center, University of California, San Francisco, CA, USA; 4 Department of Neurological Surgery, University of California, San Francisco, CA, USA

**Keywords:** Cognition, epilepsy, semantics, brain, neuropsychology, crowdsourcing

## Abstract

**Objective:**

Brain areas implicated in semantic memory can be damaged in patients with epilepsy (PWE). However, it is challenging to delineate semantic processing deficits from acoustic, linguistic, and other verbal aspects in current neuropsychological assessments. We developed a new Visual-based Semantic Association Task (ViSAT) to evaluate nonverbal semantic processing in PWE.

**Method:**

The ViSAT was adapted from similar predecessors (Pyramids & Palm Trees test, PPT; Camels & Cactus Test, CCT) comprised of 100 unique trials using real-life color pictures that avoid demographic, cultural, and other potential confounds. We obtained performance data from 23 PWE participants and 24 control participants (Control), along with crowdsourced normative data from 54 Amazon Mechanical Turk (Mturk) workers.

**Results:**

ViSAT reached a consensus >90% in 91.3% of trials compared to 83.6% in PPT and 82.9% in CCT. A deep learning model demonstrated that visual features of the stimulus images (color, shape; i.e., non-semantic) did not influence top answer choices (*p* = 0.577). The PWE group had lower accuracy than the Control group (*p* = 0.019). PWE had longer response times than the Control group in general and this was augmented for the semantic processing (trial answer) stage (both *p* < 0.001).

**Conclusions:**

This study demonstrated performance impairments in PWE that may reflect dysfunction of nonverbal semantic memory circuits, such as seizure onset zones overlapping with key semantic regions (e.g., anterior temporal lobe). The ViSAT paradigm avoids confounds, is repeatable/longitudinal, captures behavioral data, and is open-source, thus we propose it as a strong alternative for clinical and research assessment of nonverbal semantic memory.

## Introduction

The human brain can retain vast amounts of long-term general knowledge in the form of concepts, associations, raw facts, and other objective data. This cognitive domain is often referred to as semantic memory, and current neuroscience frameworks propose that its neural substrates are distributed throughout the brain in distinct cortical “semantic hub” regions. For instance, stronger blood-oxygen-level-dependent activity detected by functional magnetic resonance imaging is present in “hub” regions during semantic processing tasks (Binder et al., 2009; Martin, 2016; McGeown et al., 2009). These regions appear to interact together to associate different concepts and types of information for long-term factual encoding and recall.

Several semantic hubs, including the anterior temporal lobe (Gesierich et al., 2012; Gorno-Tempini et al., 2011), angular gyrus (Ben-Zvi Feldman et al., 2023), and precuneus (Valles-Salgado et al., 2022), among other regions (Binder et al., 2009), are implicated in clinical syndromes evidencing semantic processing impairments. These syndromes include semantic variant primary progressive aphasia (svPPA) by definition, as well as Alzheimer’s dementia, and traumatic brain injury (Gorno-Tempini et al., 2011; McGeown et al., 2009; McWilliams & Schmitter-Edgecombe, 2008; Staffaroni et al., 2021). Patients with epilepsy (PWE), especially temporal lobe epilepsy, often have damaged circuits in some of these same regions as well. However, whether PWE suffer from semantic processing deficits is an ongoing question. The literature is mixed with some studies reporting general semantic processing deficits in temporal lobe epilepsy (Barrett Jones et al., 2022; Giovagnoli, 1999; Jaimes-Bautista et al., 2015; Jensen et al., 2011) whereas others suggest strong dependence on the sublobar regions affected by the epilepsy (Anna Rita Giovagnoli et al., 2005; Smith & Lah, 2011).

It is challenging to delineate impairments in semantic memory, including in PWE, because of the reliance on language-based (verbal) tests. Common clinical neuropsychological tests engaging long-term semantic knowledge processing include semantic fluency, auditory naming (Hamberger & Seidel, 2003), the Boston Naming Task, the Frontotemporal Lobar Degeneration Module and Uniform Data Set Multilingual Naming Tests (Staffaroni et al., 2021). However, these tasks require comprehension and/or speaking of words, making it difficult to delineate semantic processing from lexical and acoustic comprehension, reading, and word-finding, which are known to be independently affected in PWE (Hamberger, 2015).

A nonverbal semantic processing task paradigm may provide further insight into whether semantic memory processing is affected in PWE. The most well-known nonverbal semantic paradigm is the Pyramids and Palm Trees (PPT) task created in 1992 (Howard, 1992). On the PPT, participants are shown a single image at the top (“stimulus”) and two images at the bottom. They must select the one (“target”) image at the bottom that is “most related” to the stimulus (the image that is less related is the “distractor”). The PPT has been adapted into new versions by other groups, such as the modified Camel and Cactus Test (CCT; (Bozeat et al., 2000; Moore et al., 2022) and other test variants (Janssen et al., 2022; Savage et al., 2013) that feature multiple improvements (e.g., color stimuli, four answer choices instead of two). However, certain features may undermine the use of the PPT and other adaptations (Janssen et al., 2022) for evaluating nonverbal semantic memory. In these tasks, performance is scored according to “intended” or “correct” answer for each trial, but a different answer may be appropriate to a given participant depending on individual context and life experiences. Thus, choosing a correct but “less popular” answer would get scored as an incorrect response, despite successful semantic memory processing, with clinical implications (e.g., misdiagnosis) and implications for research (trial accuracy misclassification). Other potential drawbacks include limited total trial numbers (statistical power considerations), stimuli sets may be proprietary (though some are posted openly (Janssen et al., 2022)) and most lack a computer interface that can track other quantitative behavioral metrics (e.g., reaction time).

We created a new version of an associative image stimulus-based behavioral task called the Visual Semantic Association Task (ViSAT), adapting the PPT/CCT paradigm to overcome these limitations. We utilized online crowdsourcing approaches to obtain probability estimates of each answer choice to aid statistical modeling, and we tested this paradigm in control participants from the community (Controls) and PWE.

## Method

### Participants

We recruited participants between ages 18 and 80 (Table 1) consisting of volunteer control participants (Controls; *N* = 24) from the community through flyers. Participants with focal/localization-related or primary generalized epilepsy conditions (PWE; *N* = 23) were recruited similarly with community flyers and through the Outpatient Epilepsy Clinic and Epilepsy Monitoring Unit at UCSF. We excluded PWE who were later deemed to have a significant medical condition that was not epilepsy (*n* = 2), and excluded Control participants (*n* = 3) due to data corruption from a computer error. Control participants were screened prior to participation and none reported a significant neurological or psychiatric disorder. Participants underwent informed consent and this study was approved by the UCSF Institutional Review Board in accordance with the Helsinki Declaration.

There were three consecutive cohorts Amazon Mechanical Turk (Mturk) workers who provided initial development and validation data as well as crowdsourced normative data for the ViSAT task (*N* = 100, *N* = 110 and *N* = 54; USA-based, Human Intelligence Tasks ratings >95%). The third cohort also provided PPT and CCT task data for comparison.

### Behavioral tasks

**Figure 1.**
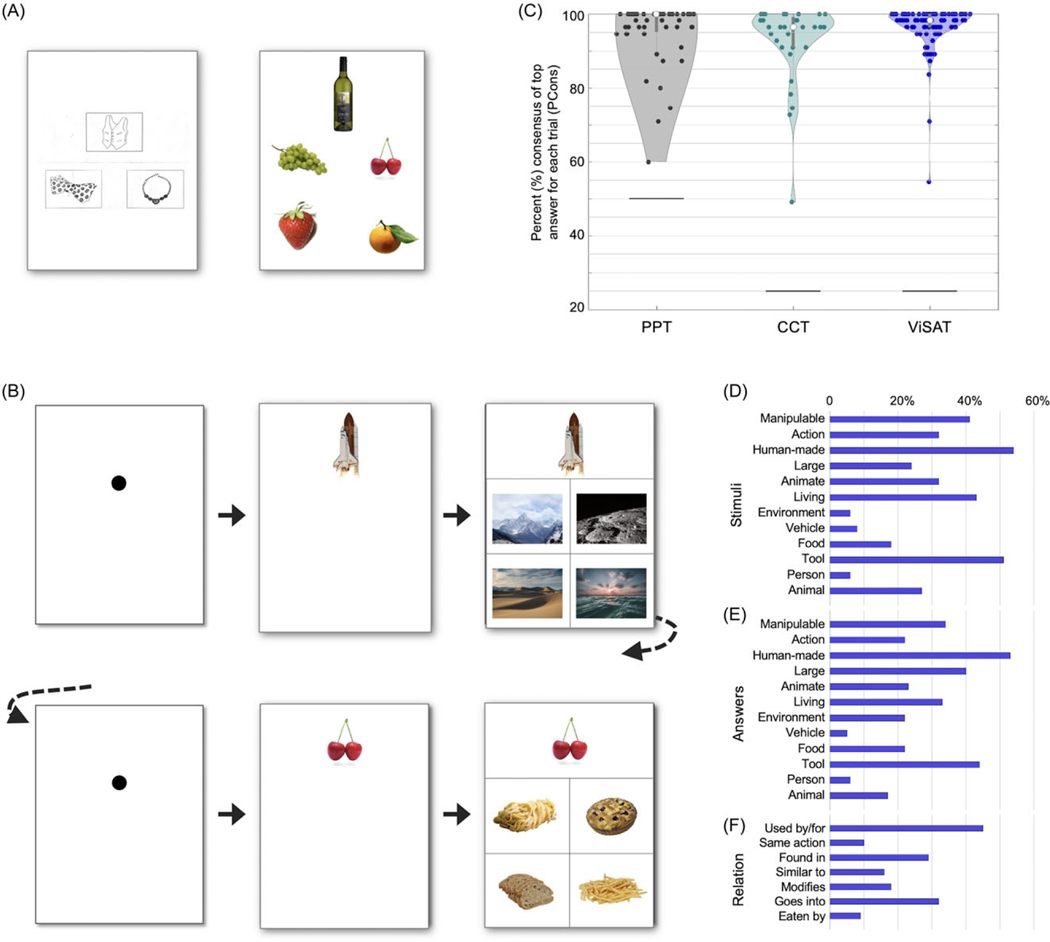
Non-verbal image-based semantic association assessments including ViSAT. **A.** Example trials from the classic PPT task (Howard, 1992) at left and the more recent modified CCT (Moore et al., 2022) at right. The layout above shows each stimulus image at the top and the answer choices below. **B.** Two example trials (rows) from the ViSAT task described in this manuscript, including fixation stage (left, 2–3 s jittered duration), stimulus stage (middle), and answer stage (right). Control and PWE participants experienced stimuli presented in isolation (middle) and advanced only after clicking it, ensuring attendance to the stimulus and enabling cognitive and behavioral time-locking of both stimulus and answer stages separately, as well as answer choice. Mturk workers experienced stimulus simultaneous with answers (right panels) in a similar manner as they did with PPT and CCT trials in A. **C.** Violin plots show distributions of the percent (%) consensus among Mturk workers (*n* = 54) of the top answer for each trial (dots) of the PPT task (*n* = 51 trials), CCT task (*n* = 32), and ViSAT task (*n* = 100). Notably, the probability of obtaining a consensus at chance (black lines) is 50% for PPT (undermining direct statistical comparison with CCT and ViSAT) and 25% for both CCT and ViSAT. Distributions illustrate a significant trend toward a higher PCons in the ViSAT compared to the CCT (*p* = 0.0488, Mann-Whitney U test). **D.** Percent of trials containing content from different semantic categories for the stimulus images for all ViSAT trials (*N* = 100). **E.** Comparable to D for trial answer choices (for trials in which there was variation of categories across trials, the Mturk consensus answer image was given precedence here). **F.** Comparable to D and E for the general semantic relationship between the stimuli to the answer choices.

The ViSAT task was adapted from concepts and similar stimuli as PPT and CCT (Figure 1A), yet with a variety of features changed. First, as opposed to the PPT, we used new color and picture images from royalty-free stock photo repositories online (pexels.com, pixabay.com, and unsplash.com). Second, to increase the potential generalizability of ViSAT across participants of all backgrounds (age, language, education, literacy levels, and socioeconomic status), we avoided religious, generation-specific, culture-specific, outdated, and potentially offensive references. Third, to decrease the confounding influences from visuospatial processing, we strived to avoid consistencies in color, size, and shape between stimuli and answers, and quantitatively compared visual feature similarity between images using a deep learning-based image attribute embedding model called ResNet-18 (He et al., 2016).

The Visual Semantic Association Task (ViSAT; Figure 1B) was administered to Controls and PWE through a user interface (UI) developed in MATLAB (Natick, MA) version R2022b. Each trial began with a centered black dot at which the participant was instructed to look (Fixation stage) with a 2–3 s interstimulus intertrial interval (duration jittered randomly). A stimulus image was then shown at the top (Stimulus stage), and once the participant clicked on this image, the four answer choices were shown below (Answer stage). The participant was instructed to click the answer most related to the stimulus in their opinion (Response stage), and the Fixation stage for the subsequent trial immediately followed.

Trials were administered in blocks of 25 trials, and each block was immediately preceded by three practice trials (always the same for each block) to ensure acclimation to the UI prior to unique trials. There are four independent blocks, for a total of 100 unique ViSAT trials, and blocks were performed either during the same session or during different sessions/days to prevent fatigue. Choices and response times were recorded by the software for later analysis. The task materials including user interface software and image stimuli are freely available on GitHub (https://github.com/Kleen-Lab/ViSATUI).

**Figure 2.**
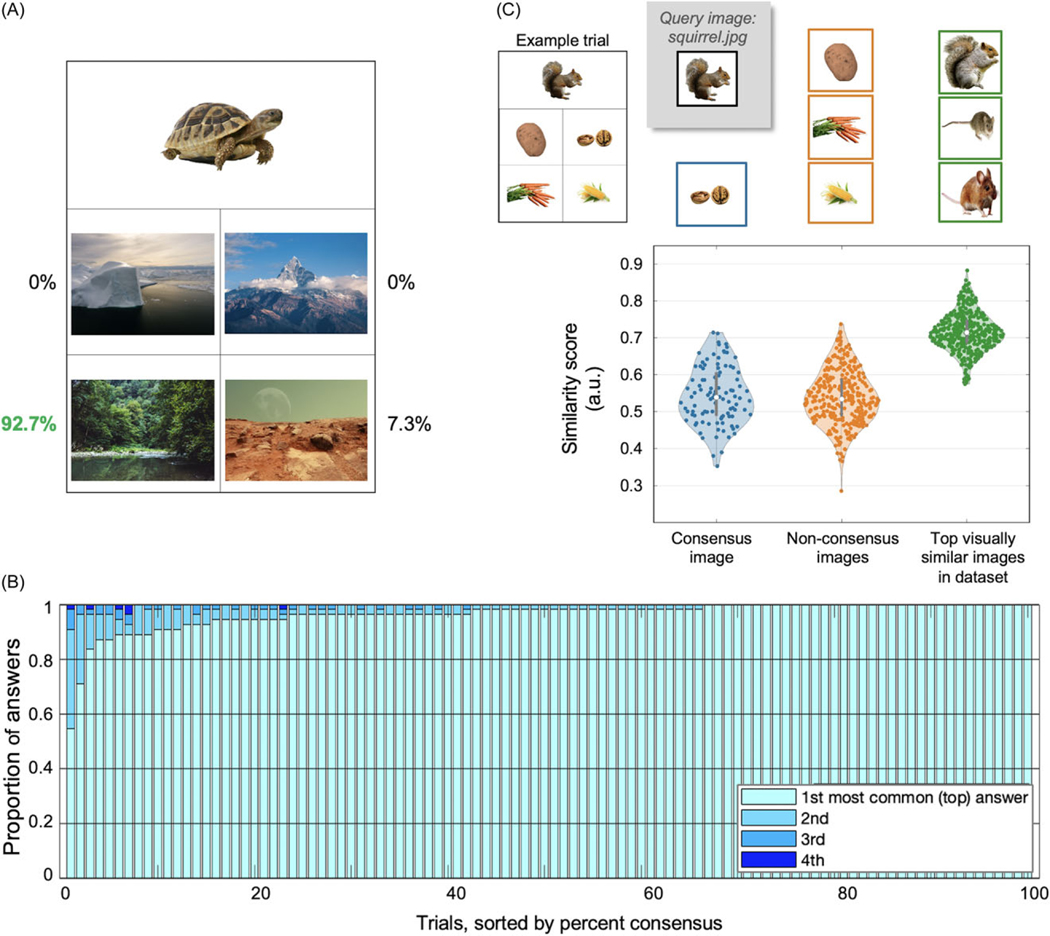
ViSAT consensus breakdown and image feature similarity. **A.** Breakdown of percent of Mturk workers who chose each answer (Pcons in green). See Supplemental Figure 1 for more detail on refinement process during ViSAT development. **B.** Breakdown of answer proportions for each trial (*n* = 100), sorted by consensus answer proportion (Pcons). The majority of trials (*n* = 92) reached a Pcons above 90%. **C.** Visual feature similarity score distributions calculated using ResNet-18 on an image2vec embedding (based on shapes, colors, textures and other features; i.e., non-semantic). Image similarity comparison scores (0 = no similarity, 1 = perfectly similar) were made between stimuli images vs. consensus answers (blue), vs. non-consensus answers (orange), and as a control the top visually similar images for each stimulus (green). Similarity scores were no different between consensus and non-consensus conditions (*p* = 0.577, two-sample *t*-test) whereas the top visually similarity scores were significantly higher than the consensus condition (*p* < 0.001).

Semantic processing and related associations can vary between participants based on factors such as personal experiences and backgrounds. Therefore, in contrast to prior approaches, we did not consider answers as “correct” or “incorrect” but instead obtained normative data and quantified the proportion of responses for each choice, convening on a “consensus” (top) answer as the “accurate” response. We used the percent consensus of the top answer (PCons, similar to percent convergence; Figure 2A) as a metric. During the development of the ViSAT task, we also obtained Mturk answer choice data to aid trial refinement (see Results). After the first and second cohorts, we revised any trials in which the PCons was<90%, adapting trials through discussion of answer choice proportions and input from a neurolinguist (J.M.J.V.) and neuropsychologist (K.C.) before running a third cohort for final crowdsourced normative data (*N* = 54). We randomly interleaved ViSAT trials (*N* = 100) with PPT (*N* = 51) and CCT trials (*N* = 35). For each trial, the single stimulus image and the answer choices (two for PPT, four for ViSAT and CCT; Figure 1A–B) were simultaneously displayed, and the answer choice for each trial was recorded. We also obtained age in years and years of formal education (cumulative; 1^st^ grade considered as year 1).

### Statistical analysis

We initially estimated our sampling size to require a minimum of 16 participants in each group to detect a 5% difference in accuracy based on Mturk group data variance (continuous endpoint from independent samples), but anticipating relatively more variability in our PWE group we increased to a target of 23 per group consistent with the upper end of sampling sizes of other recent studies using a similar previous paradigm (Janssen et al., 2022; Savage et al., 2013). Comparisons between groups or conditions were performed using two-sample t-tests for normally distributed data or Wilcoxon signed rank tests for skewed distributions. We used linear mixed effect models to model the effects of participant group and PCons (fixed effects) on reaction time (transformed using natural log) and separately on accuracy relative to PCons, adjusted with individual participants as a random effect. Correlations were performed using Spearman’s rank correlation coefficients to account for skewed data including potential ceiling/floor effects.

## Results

### Participants

Participants in all groups ranged from 19 to 80 years old (medians 38, 37, 29 for Mturk, Control, and PWE groups, respectively; Table 1). The number of years of education (capped at 20 years for analysis herein), including grade school, ranged from 6 to 20 years (capped; medians 14, 18, and 13 for Mturk, Control, and PWE groups).

### Development: Mturk-derived PCons and image analysis

Following initial creation of 100 trials as described in the Methods, the median PCons value was 95.5% (range: 37.3–100%, *n* = 110 Mturk workers). After review and adjustment/refinement of problematic elements (eg., visual feature similarity, ambiguity) for trials with<90% consensus, the median PCons for the second version was 95.5% (range: 59.1–100%; *n* = 100 Mturk workers). Following another similar round of refinements, the final version of ViSAT showed a median PCons of 98.2 (range: 54.5–100%; *n* = 54 Mturk workers). The distributions of PCons for each trial across the three versions are shown in Supplemental Figure 1.

The PCons data for the final ViSAT version was derived from this latter Mturk cohort. The PCons for all trials as well as the consensus breakdowns among the four answer choices for each trial are shown in Figure 2B. The vast majority (91.0%) of trials had a PCons >90% compared to 84.3% in PPT and 81.3% in CCT, in line with the goal of minimizing falsely incorrect answer choices while still maintaining a range of PCons to adjust control for trial difficulty. The four sets of 25 trials were counterbalanced such that there was no statistical difference in PCons across them (*p* = 0.806, Kruskall–Wallis test).

The final trial set had a diverse makeup of semantic categories of the images, and of semantic relations between the stimuli and answers (Figure 1D). To evaluate whether the similarity of visual features (non-semantic) differed between top PCons answers and non-consensus (2^nd^–4^th^ most common) answers despite our efforts to minimize these influences, we used a deep learning model (ResNet-18 and image2vec embedding) to evaluate pairs of images. We compared a given stimulus image versus its corresponding consensus answer, or versus its non-consensus answers, and found no significant difference between these scenarios (*p* = 0.577, two-sample *t*-test; Figure 2C). For comparison, the similarity scores of a given stimulus image to its top 4 visually similar images (from the entire ViSAT trial image dataset) were significantly higher compared to the consensus and non-consensus answer images (both *p* < 0.001, two-sample t-tests).

### Validation: ViSAT, PPT, and CCT in Mturk cohort

We next compared the distributions of PCons of the ViSAT with previously established clinical tasks for nonverbal semantic memory using image association (PPT, CCT), shown in Figure 1C. Notably, in the PPT task the PCons as a metric is relatively inflated due to having only two answer choices (chance 50%), compared to four in CCT and ViSAT (chance 25%), undermining direct statistical comparison. The ViSAT had a higher PCons compared to the CCT (*p* = 0.0488, Mann–Whitney *U* test).

### Validation: PCons between groups

**Figure 3.**
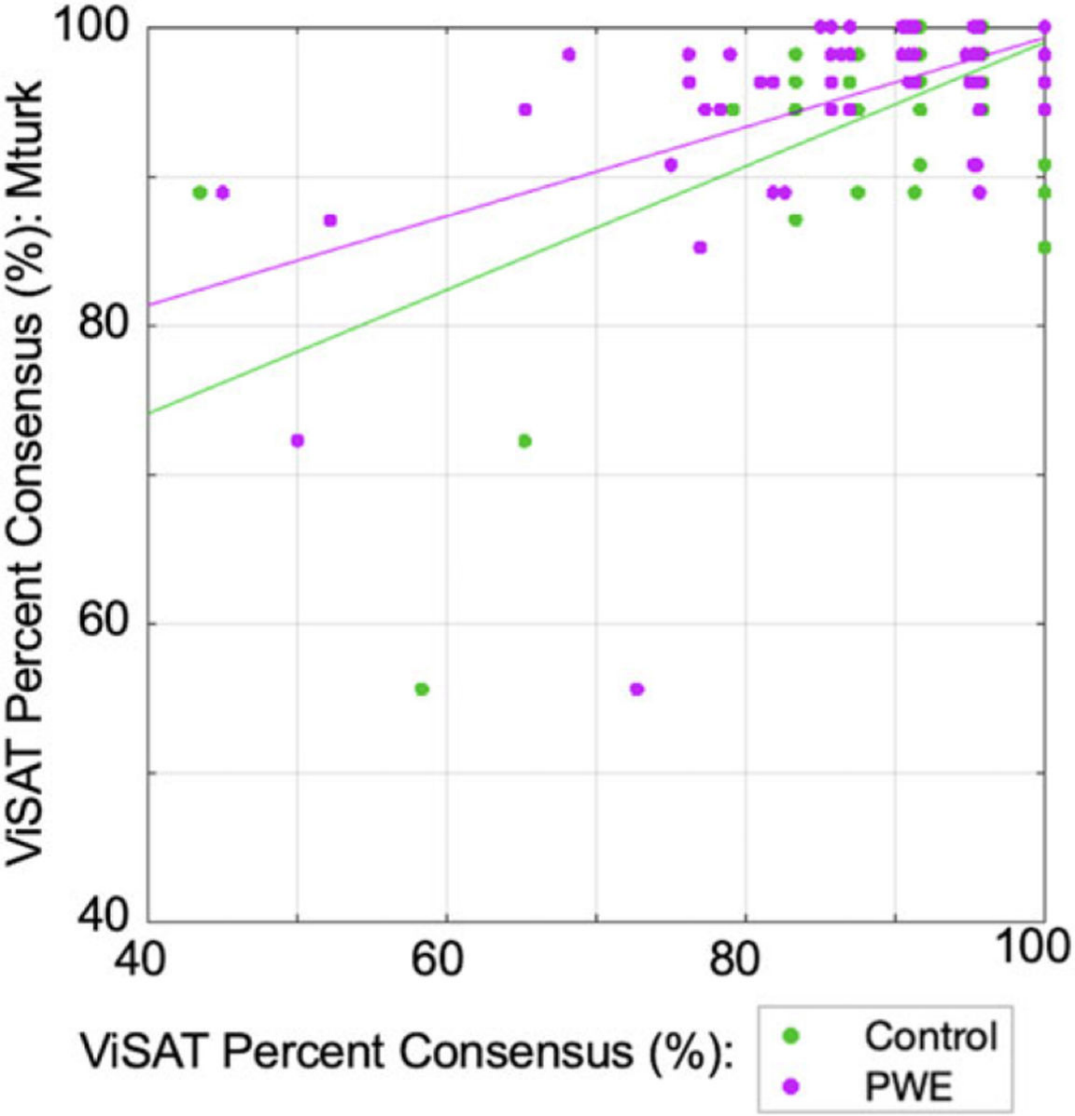
Trial-level correlation data between the percent consensus for ViSAT (Mturk, *y*-axis) versus healthy control participants (green; *r* = 0.541, *p* < 0.001, Spearman) and participants with epilepsy (magenta; *r* = 0.522, *p* < 0.001, Spearman).

To evaluate whether performance generalized across groups, we evaluated the ViSAT PCons derived from Control or PWE groups versus Mturk workers, confirming positive correlations in both scenarios (both *p* < 0.001, Spearman; Figure 3). In light of this result and having demonstrated above that the PCons for ViSAT was comparable in practice to the established PPT and CCT tests (Figure 1C), we henceforth designated PCons (top) answer as the “correct” answer for a given trial (i.e., consensus-based) and used the ViSAT PCons value (%) as a difficulty index for subsequent analyses.

### Performance between groups: Accuracy

**Figure 4.**
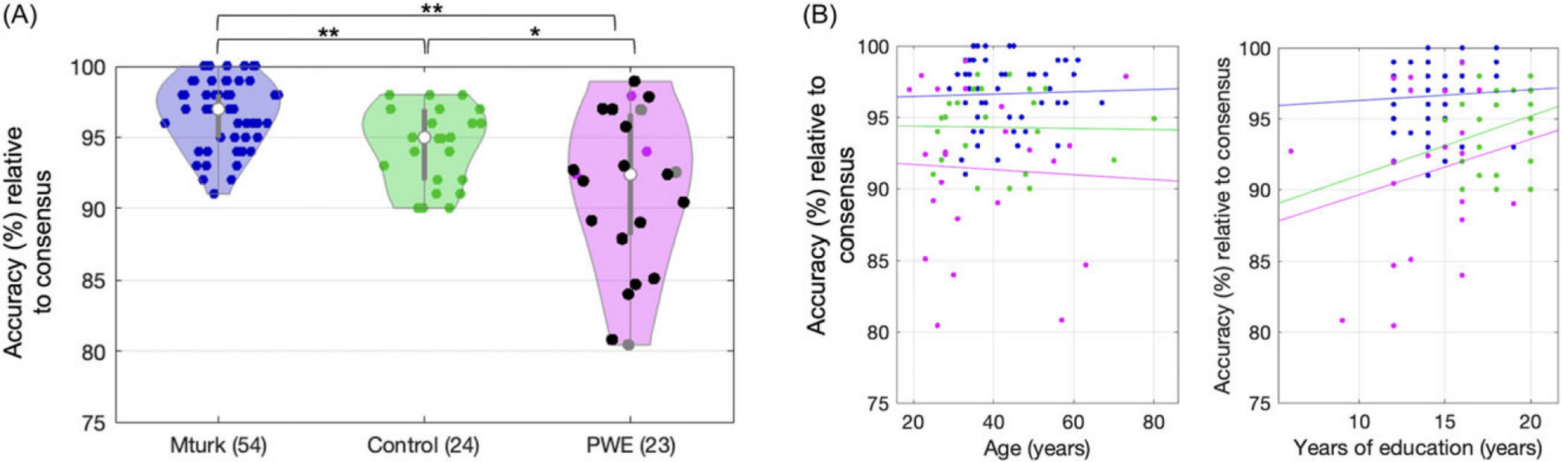
ViSAT accuracy for individual subgroups. **A.** Violin plots show distributions of accuracy for each group, derived from the top (consensus) answers from Mturk normative data designated as the correct choices (dots=individual participants; white dots=medians; grey lines=interquartile ranges; black dots=temporal lobe(s) involved in seizure onset zone; grey dots=primary generalized epilepsy). The Mturk group showed significantly higher percent accuracy (relative to consensus; PCons) than the Control and PWE groups, and the PWE group showed lower PCons than the Control (**p < 0.001, *p = 0.019; two-sample t-tests). **B.** Correlation scatterplots show lack of correlation between individual accuracy versus age (left) or years of education (right) among any group (colors=groups as in A; *p* > 0.05 for all, Spearman; least squares lines shown for illustrative purposes only).

ViSAT accuracy (percent correct relative to PCons) was significantly different between the Mturk (mean accuracy 96.6%) and Control (94.4%) groups (*p* < 0.001, two-sample *t*-test), and between the Mturk and PWE (91.4%) groups (*p* < 0.001), though the trial delivery conditions were notably different (see Methods). PWE accuracy was lower than Controls (*p* = 0.0186), and those with a seizure onset zone in the temporal lobe(s) appeared to be particularly affected (Figure 4A) though we were underpowered to assess this further. As anticipated, there was a lack of correlation between individual accuracy versus age, or versus years of education, among any group (*p* > 0.05 for all, Spearman; Figure 4B) by design (see Discussion).

### Performance between groups: Reaction time

**Figure 5.**
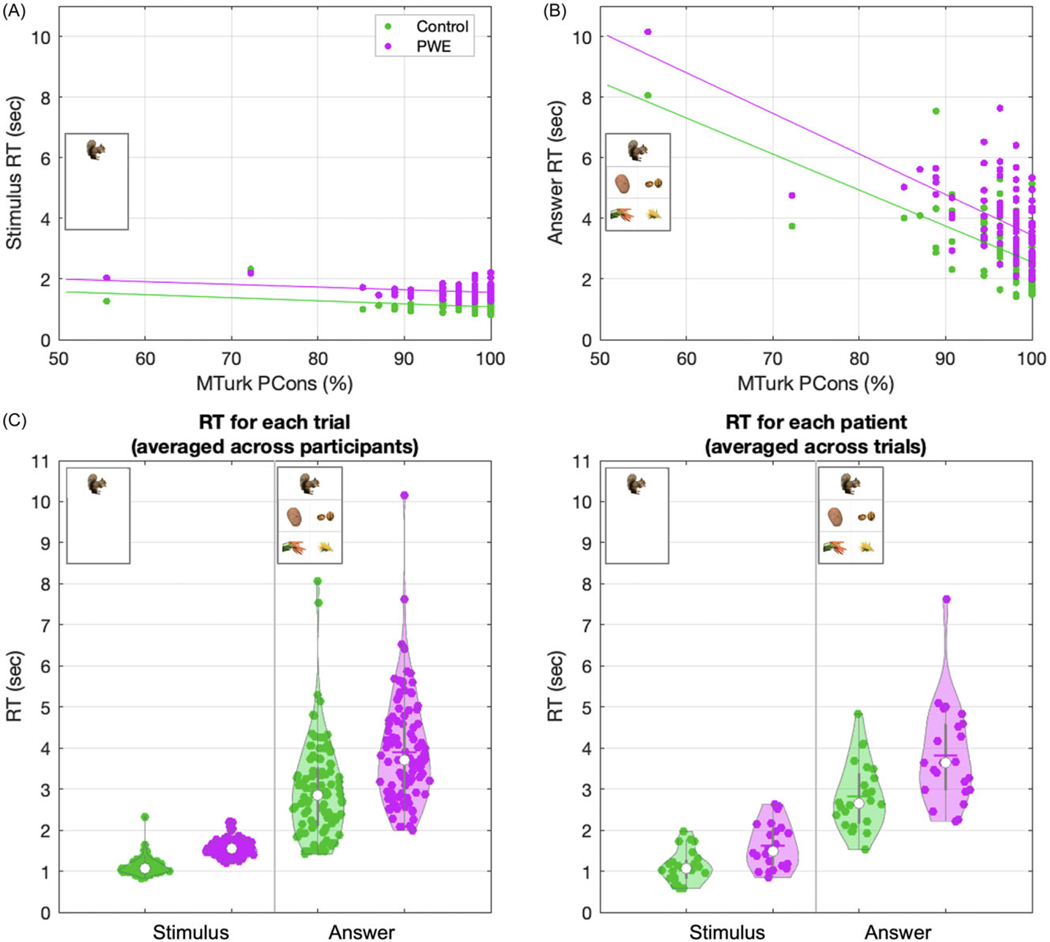
Response times in the ViSAT task. **A.** Trial-level correlation data (individual data points averaged across patients in each group) between the Mturk PCons versus response times to click the stimulus image showing no relation for either Control and PWE participants (left panel; *p* > 0.05 for both groups, Spearman). **B.** Increasing PCons (i.e., easier trials) were related to a faster response time for selecting an answer image (right panel; *r* = −0.561 and *p* < 0.001 for Control, and *r* = −0.546 and *p* < 0.001 for PWE, Spearman). **C.** Left panel shows distributions of response times for individual trials (averaged across all participants for each group), and right panel shows same data in distributions for individual patients (averaged across all trials for each participant). Longer response times were shown for the answer images than the stimulus images, and PWEs with epilepsy had longer response times in general (both *p* < 0.001, fixed effects from linear mixed effects model). Finally, an interaction was noted where, relative to Controls, PWEs with epilepsy took significantly longer to click the answer images than the stimulus (*p* < 0.001).

We next examined response time (RT; time taken to click the stimulus or answer image after being presented) as a dependent variable. Average RT for individual trials (averaged across patients) and individual patients (averaged across trials) are shown in Figure 5A–B. The PCons for individual trials did not correlate with RT for stimuli (*p* > 0.05, Spearman). However, there was a strong negative correlation with RT for answer choices, i.e., during semantic association processing (*p* < 0.001 for both Control and PWE groups, Spearman; Figure 5B).

RTs had positively skewed distributions hence the use of non-parametric rank correlations above. For mixed-effect modeling we transformed this data comparing square root and natural log conversions. We convened on the square root transform (RT_sqrt_) after confirming a comparatively better fit for subsequent linear mixed-effect models (*p* < 0.001, log-likelihood ratio). We modeled RT_sqrt_ with individual as a random effect and used fixed effects of group (Control or PWE), condition (stimulus or answer stage), and PCons to adjust for trial difficulty:

### RT_sqrt_ ∼ 1 + group + condition + group*condition + PCons + (1|participants)

Similar to above, PCons was inversely related to RT_sqrt_ (*p* < 0.001, OR −0.017, CI −0.19 to −0.16, linear mixed effects model). RT_sqrt_ to click the answer choice was significantly longer than the time to click the stimulus (*p* < 0.001, OR −0.739, CI −0.764 to −0.713). The Control group had shorter RT_sqrt_ than the PWE group (*p* < 0.001, OR −0.288, CI −0.408 to −0.167). An interaction between group and RT suggested PWE took more time to choose an answer than to click the stimulus (*p* < 0.001) compared to Controls (Figure 5C–D).

## Discussion

This study evaluated semantic processing in PWE using a novel image association task that elicited retrieval of general long-term knowledge, specifically factual associations between items and/or contexts. We aimed to understand whether PWE have potential deficits in semantic processing that transcend acoustic, linguistic, verbal or other language-related functions which are known to be independently affected in epilepsy (Corcoran & Thompson, 1993; Hamberger, 2015; Kleen et al., 2012). Thus as opposed to most semantic neuropsychological testing paradigms that are confounded by expressive language skills, we designed and adapted a task free of verbal requirements. We noted performance impairments in both choosing the correct answer (accuracy) and the time taken to choose it (response time) when compared to Control participants.

PWE showed significantly longer RTs in general (both stimulus and answer) compared to controls. We surmise this result could be partially explained by the effect of anti-seizure medications on cognition (Eddy et al., 2011), and/or an increased prominence of psychomotor slowing among PWE (Garcia-Ramos et al., 2018; Sung et al., 2013). Such influences would be challenging to disentangle, requiring much larger studies (e.g., with statistical power to adjust for type and dosing of medications and/or baseline psychomotor slowing). Crucially, there was a significant interaction: relative to Controls, PWE groups took significantly longer to click the answer choice than they did to click the stimulus (interaction between condition and group; Figure 5). In other words, when adjusting for generally slowed RT, PWE required a compounded amount of additional time relative to Controls to respond in the answer stage of the task. This pattern is consistent with impaired semantic processing considering the additional associative processing required to select the target answer.

Patients with focal epilepsy may have dysfunctional brain areas overlapping with the seizure onset zone(s) that are part of the putative substrates of semantic memory processing (Binder et al., 2009; Gesierich et al., 2012; Gorno-Tempini et al., 2011; Martin, 2016). Atrophy patterns and associated clinical deficits in svPPA implicate the anterior temporal lobe in semantic processing (Gesierich et al., 2012; Gorno-Tempini et al., 2011). Temporal lobe epilepsy is the most common epilepsy (Téllez-Zenteno & Hernández-Ronquillo, 2012; Wiebe, 2000). and often these patients have dysfunction localized to the anterior temporal lobe, a known heteromodal hub for semantic memory processing and integration (Abel et al., 2015; Forseth et al., 2018). In fact, there is a growing body of evidence that even medial temporal lobe structures, including the hippocampus which is perhaps the most commonly implicated seizure onset focus in epilepsy (Téllez-Zenteno & Hernández-Ronquillo, 2012). may play a larger potential role in semantic memory than previously anticipated (Bayley & Squire, 2005; Duff et al., 2019). Despite these connections it has been unclear whether semantic memory is truly affected in focal epilepsy or if deficits may have been conflated with verbal memory impairments which are commonly affected in focal epilepsy (Hamberger, 2015). Here we demonstrate that nonverbal semantic processing indeed appears to affected. While we cannot rule out the possibility of silent speech, participants were instructed to avoid talking internally or out loud, and our careful curation of task stimuli devoid of words strived to make sure that the results here were independent of verbal influences.

Our comparison of the PCons across the classic PPT task (Howard, 1992) and the more recently modified CCT (Moore et al., 2022) underscored some intentions of our redesign into the ViSAT task. The results across 54 cognitively normal individuals showed only 82.9% of trials in CCT and 83.6% of trials in PPT in which more than 90% gave the same answer. The latter is particularly striking since performance at chance is 50% in the PPT task (only two answer choices), and in fact some PPT trials had a PCons as low as 60%. Put another way, up to 40% of Mturk workers who presumably do not have a neurological condition (Figure 4A) chose a PPT answer that was not the consensus answer (Figure 1C). These numbers suggest a substantial and previously undescribed risk for falsely-incorrect trials (and thus misdiagnosis) despite choosing a potentially plausible (non-consensus) answer in earlier paradigms. These differences may reflect differences in life experiences, or demographic or cultural experiences. We repeatedly tailored ViSAT trials until the PCons was >90% consensus for >90% of trials to address this issue, and we demonstrated that this approach is not undermined by ceiling effects as we effectively delineated differences between groups. Furthermore, the constrained residual variance in PCons remains a strong metric of difficulty that is important in statistical modeling of performance (Figures 4 and 5).

Our study encompasses several strengths. We adapted our task substantially from prior versions to improve generalizability, delivery logistics, and longitudinal use. To increase statistical power and signal-to-noise ratio we created a large number of trials (*n* = 100) and used four answer choices (Janssen et al., 2022) as opposed to two in PPT. The ViSAT trials are divided into four 25-trial sets with similar difficulty across them (see Results) to accommodate statistical power needs and aid longitudinal testing. To improve the variety and generalizability of trial materials, we used color picture images drawn from royalty-free stock photo repositories, and avoided religious, generation-specific, outdated, or potentially offensive references. We ensured that by nature no text is required in this nonverbal task. We also minimized visuospatial pattern confounds by reducing shared characteristics (color, size, shape) between stimuli and answer choices, and confirmed this quantitatively using a deep learning image comparison model. Lastly, we strived to use images that were approachable across education levels and languages. Corroborating these efforts to minimize bias, there was no correlation of ViSAT accuracy with age or with years of education (Figure 4B).

Limitations of our study include that the three groups consist of predominantly White and Asian individuals, limiting generalizability to other race and ethnicity groups. The Mturk and Control groups had relatively more years of education, though by design through trial image curation there was no correlation of this metric to performance. The ViSAT is tested here in predominately English speakers, and future studies on non-English speakers are needed to evaluate cross-cultural applicability. Importantly, demographic, cultural, and language differences were a major influence on our design process for this nonverbal task and so we anticipate that no significant task modifications should be necessary prior to direct comparison across different languages in future research and potentially clinical settings.

The majority of PWE in our study had focal epilepsy involving the temporal lobe (Supplementary Table 1, Figure 4A), and while our results may therefore be most relevant for temporal lobe epilepsy, this group had variable characteristics at the individual level (Supplementary Table 1). Some of these factors could plausibly influence semantic memory performance including epilepsy type and localization/lateralization of the seizure onset zone(s), which could overlap with, and cause dysfunction in, key semantic processing regions (e.g., anterior temporal lobe). Additional factors such as type and dosing of the numerous different anti-seizure medications, and seizure frequency, could plausibly affect accuracy and reaction time. These variable factors may have driven the wider variability (distributions) in performance data relative to Controls (Figures 4A and 5). While we were relatively underpowered to assess these factors in more detail the results herein are compelling for fueling future larger investigations into epilepsy-related semantic memory dysfunction, both in our own work and facilitated for others by our freely available ViSAT paradigm.

We propose our ViSAT task as a step forward in the nonverbal evaluation of semantic memory processing. This task carefully avoids language to minimize verbal, lexical, and acoustic influences, providing a more focused and versatile assessment of semantic processing function. The ViSAT may be a helpful tool for future studies on the anatomic localization of specific semantic category domains (Binder et al., 2009; Gesierich et al., 2012; Hamberger et al., 2007). Since the ViSAT was designed to avoid previous major confounds, is repeatable/longitudinal, measures behavioral data, and is open-source, we propose it as a strong alternative for clinical assessments of nonverbal semantic memory function and research investigations of normal and abnormal semantic processing.

## Supplementary Material

1

## Figures and Tables

**Table 1.** Demographic information for all groups. Age and Education expressed as median, range in parenthesis. Gender, race, and ethnicity expressed as percentage (y = year, M = male, F = female, NB = nonbinary, AIAN = american indian/alaska native, A = asian, B = black, M = more than one race, O = other, W = white, H = hispanic, NH = non-hispanic)

	Age (y)	Education (y)	Gender (%; M,F,NB)	Race (%; AIAN,A,B,M,O,W)	Ethnicity (%, H,NH)

Mturk	38 (29–67)	14 (12–22)	51.9, 44.4, 3.7	0.0, 3.7, 1.9, 0.0, 5.6, 88.9	7.4, 92.6
Control	37 (25–80)	18 (14–33)	66.7, 33.3, 0	0.0, 45.8, 4.2, 0.0, 16.7, 33.3	16.7, 83.3
PWE	30 (19–73)	13 (6–19)	63.2, 36.8, 0	4.3, 13.0, 4.3, 8.7, 26.1, 43.5	43.5, 56.5
