# Supplementary material for: Development and validation of a nonverbal consensus-based semantic memory paradigm in patients with epilepsy
Source: J Int Neuropsychol Soc. Author manuscript; Available in PMC 2024 Oct 18. (PMC11473708; doi:10.1017/S1355617724000158)
Supplement: 1 [file NIHMS1976815-supplement-1.docx]

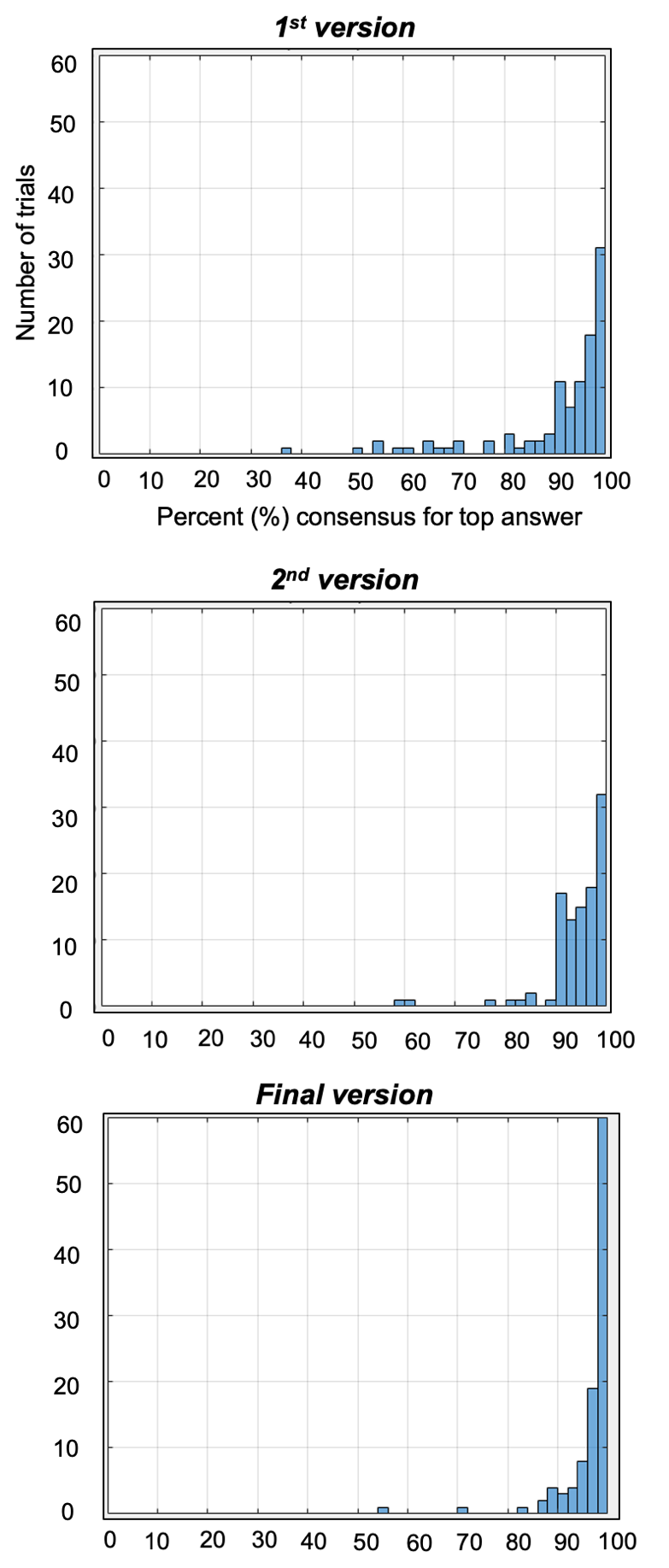


***Supplemental Figure 1****. ViSAT refinement process. Percent consensus (PCons) distributions of all 100 trials (and 3 practice trials) from the earlier versions (top and middle) and the final version (bottom), showing general increase in percent consensus.*

| **PWE ID #** | **Etiology** | **Lateralization/ localization** | **Temporal involved in SOZ** | **Seizure Frequency** | **Age of Onset (y)** | **Duration of epilepsy (y)** | **# ASMs** | **# prior ASMs** |
| --- | --- | --- | --- | --- | --- | --- | --- | --- |
| **1** | Focal | L frontotemporal | Yes | Yearly | 31 | 10 | 2 | 4 |
| **2** | Primary generalized | Primary generalized | No | Weekly | 12 | 14 | 2 | 4 |
| **3** | Focal | L mesial temporal | Yes | Yearly | 25 | 8 | 1 | 0 |
| **4** | Focal | Bilateral frontotemporal | Yes | Weekly | 18 | 7 | 3 | 3 |
| **5** | Focal | L frontotemporal and/or medial temporal | Yes | Weekly | 68 | 5 | 1 | 1 |
| **6** | Primary generalized | Primary generalized | No | Daily-weekly | 0 | 29 | 4 | 0 |
| **7** | Focal | L frontal | No | Monthly | 14 | 13 | 2 | 5 |
| **8** | Focal | R posterior temporal | Yes | Monthly | 13 | 11 | 3 | 1 |
| **9** | Focal | R frontocentral/ frontotemporal | Yes | Weekly-monthly | 13 | 13 | 3 | 2 |
| **10** | Focal | L mesial temporal | Yes | Weekly-monthly | 19 | 3 | 3 | 3 |
| **11** | Focal | R mesial temporal | Yes | Daily | 20 | 36 | 2 | 3 |
| **12** | Focal | L temporal | Yes | Monthly | 27 | 2 | 2 | 1 |
| **13** | Primary generalized | Primary generalized | No | Daily | 15 | 4 | 2 | 2 |
| **14** | Focal | Bilateral temporal | Yes | Weekly | 39 | 2 | 1 | 6 |
| **15** | Focal | L frontotemporal | Yes | Monthly | 57 | 2 | 2 | 1 |
| **16** | Focal | Bilateral temporal (suspected) | Yes | Daily-weekly | 34 | 15 | 3 | 1 |
| **17** | Focal | L frontocentral | No | Monthly | 20 | 2 | 2 | 1 |
| **18** | Focal | L basal temporal | Yes | Daily | 25 | 6 | 3 | 0 |
| **19** | Focal | R anterior temporal | Yes | Monthly | 52 | 3 | 1 | 2 |
| **20** | Focal | Bilateral mesial temporal | Yes | Monthly | 21 | 3 | 3 | 3 |
| **21** | Focal | Bilateral medial frontal/cingulate | No | Weekly | 27 | 16 | 1 | 4 |
| **22** | Focal | R frontotemporal | Yes | Weekly | 15 | 18 | 2 | 4 |
| **23** | Focal | Bilateral medial temporal | Yes | Daily | 39 | 24 | 4 | 9 |

***Supplemental Table 1****. Individual-level demographic and clinical information for PWE. Abbreviations: PWE, patients with epilepsy, y, year; R, right, L, left.*
